# Rationale for adjuvant measures in musculoskeletal diseases

**DOI:** 10.1186/1471-2474-14-S2-O10

**Published:** 2013-05-29

**Authors:** Ulf Gast

**Affiliations:** 1 Centre for Muscle and Bone Research, Charité Universitätsmedizin Berlin, Berlin, Germany

## 

Bone and muscle are dynamic tissues. Muscle adapts to stimuli above thresholds (energetic emptying > exhaustion). Wolff’s law states that structural bone adaptation is driven by the experienced bone strains. Osteocytes within our bones regulate bone formation and degradation in response to mechanical stimuli. The largest strains emerge from muscle contractions. A lot of diseases are associated with secondary muscle weakness (sarcopenia) and reduced bone density (osteoporosis). Both deficits cause an increase in fall incidence. About every 4^th^ fall results in fracture. Patients after fractures become more and more immobile. Necessary stimuli decrease further. It comes to progressive deconditioning, whereby the vicious circle is complete, because it results in decreasing muscle cross-sectional area as well as bone strength. Accordingly, therapy concepts have to focus on maintenance and increasing muscle force and power. An established method is intensive resistance exercise training aimed to hypertrophy. Also the training program must ensure that forces reach the minimal effective strain and leads to bone remodelling. High-load resistance exercises effectively increase muscle and bone at the same time.

